# Associations between body mass index and lung function using Z-scores: a nonlinear relationship and machine learning classification modeling

**DOI:** 10.3389/fphys.2025.1706759

**Published:** 2025-11-17

**Authors:** Wei Feng, Fei Lu, Jiangjiang Liu, Yu Zhang, Shiyu Shen, Haitao Ma

**Affiliations:** Department of Thoracic Surgery, The Fourth Affiliated Hospital of Soochow University, Suzhou, China

**Keywords:** BMI, lung function, NHANES, GLI-global equations, machine learning

## Abstract

**Introduction:**

This study systematically investigated the relationship between body mass index (BMI) and lung function, incorporating Z-scores, thereby offering a novel approach to lung function management.

**Methods:**

Data from the National Health and Nutrition Examination Survey (NHANES, 2007–2012) were utilized, encompassing composite measures of lung function, diet, BMI, smoking history, dust exposure, heart failure, asthma, chronic bronchitis, tuberculosis, a history of thoracic surgery and other relevant covariates. Lung function Z-scores were calculated, and their associations were evaluated using multiple linear regression, logistic regression, and restricted cubic spline models. A total of 12,783 participants were included, with participants categorized into four groups based on forced expiratory volume in one second (FEV1) Z-scores, forced vital capacity (FVC) Z-scores and FEV1/FVC Z-scores: the Z1group, representing the normal lung function group (n = 10,760), the Z2 group, representing the obstructive ventilatory defect group (n = 1,300), the Z3 group, representing the restrictive ventilatory defect group (n = 597), and the Z4 group, representing the mixed ventilatory defect group (n = 126). Subgroup analyses were also performed. We captured the complex relationships between BMI and lung function by developing 22 derived features, employing the Synthetic Minority Over-sampling Technique (SMOTE) to address class imbalance, and training and comparing seven machine learning algorithms.

**Results:**

Among 12,783 participants (mean age 46 years, 51% male), 10,760 had normal lung function, 1,300 had obstructive ventilatory defect (OVD), 597 had restrictive ventilatory defect (RVD), and 126 had mixed defect. BMI demonstrated opposing associations with ventilatory defects: higher BMI was inversely associated with OVD risk (Q4 vs. Q1: OR = 0.532, 95% CI 0.418–0.678, P < 0.0001), but positively associated with RVD risk (Q4 vs. Q1: OR = 2.900, 95% CI 2.708–4.048, P < 0.0001). Restricted cubic spline analysis revealed a U-shaped relationship for RVD, with a threshold at 26.39 kg/m^2^. Machine learning models confirmed BMI-related features as the most important predictors, accounting for >32% of total feature importance.

**Conclusion:**

This study reveals differential and opposing associations between BMI and ventilatory impairment phenotypes, with higher BMI inversely associated with obstructive defects but positively associated with restrictive defects. Moreover, strong correlations were validated through extensive adjustments and machine learning models.

## 1 Introduction

Lung function impairment represents a substantial global health burden, with chronic obstructive pulmonary disease (COPD) alone affecting millions of individuals worldwide and restrictive ventilatory defects linked to various systemic conditions (Raher et al., 2009). Body mass index (BMI) has emerged as an important determinant of respiratory health (Zhu et al., 2021), yet the relationship between BMI and lung function remains complex and potentially phenotype-specific. However, prior studies have often been limited by the use of traditional lung function metrics (Jones and Nzekwu, 2006; Tang et al., 2022).

In 2021, the Z-score method was introduced by the American Thoracic Society (ATS) and the European Respiratory Society (ERS) as an improved approach to assess airflow limitation, superseding traditional metrics such as percent forced expiratory volume in one second (FEV1%) and the FEV1 to forceful lung volume ratio (FEV1/FVC). This method utilizes Z-scores to better stratify the severity of lung function impairment while minimizing biases associated with gender, age, height, and ethnicity, and aligns more effectively with the ATS/ERS severity classification (Stanojevic et al., 2022). Therefore, this study aims to leverage the Z-score framework to systematically investigate the nuanced associations between BMI and distinct phenotypes of ventilatory impairment in a large, nationally representative cohort.

Obesity has emerged as a risk factor for numerous diseases globally (Ng et al., 2014), with several studies indicating its impact on lung function (Santana et al., 2001; Sutherland et al., 2008). A cohort study involving 22,743 participants demonstrated a positive correlation between body mass index (BMI) and lung function, measured by FVC and FEV1 (Svart et al., 2020). Furthermore, some scholars argue that both being underweight and severely obese can lead to impaired lung function (Tang et al., 2022). However, the relationship between BMI and lung function remains complex and potentially disease-specific. Evidence suggested that BMI may have differential associations with various respiratory conditions. Studies in chronic obstructive pulmonary disease (COPD) have shown that while BMI is associated with exercise capacity, it may not adequately reflect disease severity or staging, with fat-free mass index (FFMI) demonstrating superior correlation with airflow obstruction parameters (Ischaki et al., 2007). Similarly, research on asthma has highlighted the limitations of BMI as a simple height-weight metric, demonstrating that body fat distribution measurements may have stronger and more specific associations with respiratory outcomes than BMI alone (Wang et al., 2024). These findings underscore the need to investigate whether BMI exhibits phenotype-specific associations with different patterns of ventilatory impairment, including obstructive *versus* restrictive defects.

However, prior studies were limited by small sample sizes, and relying solely on predicted FEV1 values or FEV1/FVC ratios to assess lung function impairment presents an overly simplistic view. This study introduces the use of Z-scores, offering a more comprehensive framework for understanding the relationship between BMI and lung function. The findings provide valuable insights that could inform the development of preventive and therapeutic strategies for lung function impairment in perioperative patients.

## 2 Materials and methods

### 2.1 Participants

**FIGURE 1:**
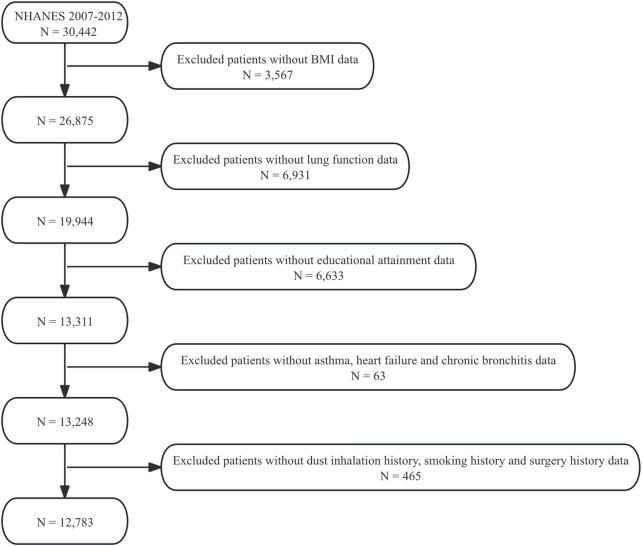
Participant selection flowchart.

This study utilized data from three National Health and Nutrition Examination Survey (NHANES) cycles (2007–2012), encompassing 30,442 participants. Individuals with missing data on BMI (n = 3,567), lung function (n = 6,931), educational attainment (n = 6,633), asthma (n = 12), heart failure (n = 33), chronic bronchitis (n = 18), dust inhalation history (n = 455), smoking history (n = 6), and surgery history (n = 4) were excluded, resulting in a final cohort of 12,783 participants (Figure 1). Complete data on sex, age, ethnicity, vitamin C intake and tuberculosis were available for the cohort.

### 2.2 Spirometry

Lung function testing in NHANES adhered to the standards set by ATS and ERS (Miller et al., 2005).

In the present study, participants were categorized into four groups based on FEV1 Z-scores, FVC Z-scores and FEV1/FVC Z-scores: normal lung function group, obstructive ventilatory defect (OVD) group, restrictive ventilatory defect (RVD) group, and mixed ventilatory defect (MVD) group (Stanojevic et al., 2022; Quanjer et al., 2014). The Z-scores account for age, gender, height, and race, providing a precise evaluation of the lung function severity. Z-scores greater than −1.645 indicate normal lung function, while those below −1.645 denote impaired lung function. Group Z1 represented the normal lung function group, with both FVC Z-scores and FEV1/FVC Z-scores greater than −1.645. Group Z2 represented the OVD group, characterised by FEV1/FVC Z-scores below −1.645, while FVC Z-scores were greater than −1.645. Group Z3 represented the presumptive RVD group, with FVC Z-scores below −1.645 and FEV1/FVC Z-scores exceeding −1.645. Group Z4 represents the MVD group, with both FVC Z-scores and FEV1/FVC Z-scores below −1.645. It is important to note that a definitive diagnosis of restrictive lung disease requires confirmation by measurement of total lung capacity (TLC), which was not available in the NHANES dataset. The following parameters were extracted from NHANES datasets for each participant: age (years), sex (male or female),height (cm, to one decimal place when available), ethnicity, measured FEV1 (L), measured FVC (L), measured FEV1/FVC (ratio). The Global Lung Function Initiative (GLI) prediction equations and specialized software were used to calculate Z-scores for the predicted FEV1 values (Quanjer et al., 2012) (www.lungfunction.org/files/InstallGLI2012_DataConversion.EXE).

### 2.3 Covariates assessment

Potential confounding variables, including age, gender, and ethnicity (Mexican American, other Hispanic, non-Hispanic white, non-Hispanic black, and other races, including multiracial), were considered in the study. Educational attainment data for adults aged 20 and older were collected through a questionnaire, with five levels ranging from less than ninth grade to college graduate or higher. Vitamin C intake over the 24 h preceding the interview was obtained from NHANES participants via the dietary interview component. Smoking history and dust inhalation history were also gathered through participant questionnaires. Furthermore, clinical characterisation encompassing heart failure, asthma, chronic bronchitis, tuberculosis, and a history of thoracic surgery was conducted.

### 2.4 Machine learning for classification and feature importance analysis

To capture complex relationships between BMI and lung function, we developed 22 derived features through advanced feature engineering:

i. BMI categorization: Six binary variables for underweight (<18.5), normal weight (18.5–24.9), overweight (25.0–29.9), and three obesity classes (Class I: 30.0–34.9, Class II: 35.0–39.9, Class III: ≥40.0).
ii. Non-linear transformations: BMI squared (BMI^2^), BMI cubed (BMI^3^), and age squared (Age^2^) to capture non-linear relationships.
iii. Interaction terms: Six interaction features including BMI × Age, BMI × Sex, BMI × Smoking, Age × Smoking, BMI × Vitamin C, and BMI × Asthma.
iv. Composite respiratory risk score: Sum of five respiratory risk factors (asthma, smoking, chronic bronchitis, dust exposure, and tuberculosis history), ranging from 0 to 5.
v. Age stratification: Three binary variables for young (<40 years), middle-aged (40–60 years), and older adult (≥60 years) groups.
vi. Health ratio indicators: BMI-to-age ratio and statistical features (mean and standard deviation of key continuous variables).

The dataset was split into training (80%, n = 10,125) and testing (20%, n = 2,532) sets using stratified sampling. Given the substantial group imbalance, we applied the Synthetic Minority Over-sampling Technique (SMOTE) to the training set, generating synthetic samples to achieve balanced group distribution (n = 8,607 per group, total n = 25,821). We developed and compared seven machine learning algorithms: Logistic Regression (cost-sensitive), Random Forest (balanced, n_estimators = 500), Gradient Boosting (n_estimators = 300, learning_rate = 0.05), AdaBoost (n_estimators = 200), Support Vector Machine (RBF kernel, cost-sensitive), Stacking ensemble (combining RF, GB, and AdaBoost), Voting ensemble (soft voting among top three models). Model performance was evaluated using 5-fold stratified cross-validation on the training set. Primary evaluation metrics included: balanced accuracy (mean of per-class recall); overall accuracy; precision, recall, and F1-score (weighted by class support); area under the ROC curve (macro-average and weighted-average); area under the precision-recall curve for each class; confusion matrix for visualizing classification patterns.

### 2.5 Statistical analyses

Data analysis followed NHANES guidelines, incorporating the complex survey design. Survey sample weights (WTINT2YR for interview data, WTMEC2YR for examination data) were applied, and 6-year combined weights were constructed by dividing 2-year weights by three for the pooled 2007–2012 cycles. Survey design variables (SDMVPSU for primary sampling units, SDMVSTRA for strata) were incorporated to obtain appropriate variance estimates. The samples were divided into four groups, and descriptive statistical analysis was performed for the OVD group and RVD group compared with the normal lung function group. The MVD group had a small sample size and therefore was not included in the primary analysis. Non-normally distributed continuous variables were expressed as medians with interquartile ranges and compared between groups using the Kruskal–Wallis H-test. Categorical data were presented as proportions and compared using the chi-square test. Participants were further categorized into four groups based on BMI (kg/m^2^). Participants were further categorized into four groups based on BMI (kg/m^2^). In the descriptive analysis comparing the OVD group with the normal lung function group, the quartile ranges were: Q1 (BMI 13.18–24.26), Q2 (BMI 24.26–27.84), Q3 (BMI 27.84–32.17), and Q4 (BMI 32.17–84.87). Similarly, in the analysis of the RVD group *versus* the normal lung function group, the ranges were: Q1 (BMI 14.20–24.47), Q2 (BMI 24.47–28.16), Q3 (BMI 28.16–32.60), and Q4 (BMI 32.60–84.87). FEV1/FEV1 Z-scores and FVC Z-scores were considered the dependent variable. Covariate adjustments were made using extended modeling techniques in multivariate logistic regression models. Three models were constructed: Model 1 (unadjusted), Model 2 (adjusted for age, gender, ethnicity and education), and Model 3 (adjusted for gender, age, ethnicity, education, vitamin C intake, smoking history, dust inhalation history, asthma, heart failure, and chronic bronchitis). Nonlinear relationships between BMI and FEV1 Z-scores were explored using restricted cubic spline regression, with nonlinearity tested via the likelihood ratio test. Subgroup analysis was conducted by categorizing variables such as age. All machine learning analyses were conducted within the open-source Python ecosystem. All statistical analyses were performed using R 4.4.2 software, with statistical significance set at p < 0.05.

## 3 Results

### 3.1 Baseline characteristics

This study included 12,783 participants with a mean age of 46 years, with males comprising 51% of the cohort. Detailed demographic characteristics of the study cohort are provided in Table 1. Participants were categorized into four groups based on FEV1 Z-scores, FVC Z-scores and FEV1/FVC Z-scores: the Z1group, representing the normal lung function group (n = 10,760), the Z2 group, representing the obstructive ventilatory defect (OVD) group (n = 1,300), the Z3 group, representing the restrictive ventilatory defect (RVD) group (n = 597), and the Z4 group, representing the mixed ventilatory defect (MVD) group (n = 126). Statistically significant differences between the groups were observed in gender, age, ethnicity, education, BMI, asthma, heart failure, chronic bronchitis, thoracic surgery history, dust exposure history, and smoking history (p < 0.05). The Z2 group was characterized by a higher median age (48.82 years), lower educational attainment, and a higher proportion of non-Hispanic White participants (51.80%). BMI levels in the Z2 group were significantly lower than those in the Z1 group (p < 0.001). There was no statistically significant difference in vitamin C intake between the Z1group and the Z2 group. Additionally, the history of asthma (24.5%), heart failure (2.4%), chronic bronchitis (7.7%), thoracic surgery (21.8%), dust inhalation (27.4%) and smoking (68.2%) was more prevalent in the Z2 group. Similarly, compared with the Z1group, the Z3 group exhibited a higher median age (50.39 years), a lower proportion of non-Hispanic White participants (41.4%), and lower educational attainment. Additionally, the history of asthma (18.6%), heart failure (5.7%), chronic bronchitis (8.4%), thoracic surgery (29.5%), dust inhalation (22.1%) and smoking (47.9%) was more prevalent in the Z3 group. In contrast, BMI levels in the Z3 group were significantly higher than those in the Z1 group (p < 0.001).

**TABLE 1:** Baseline characteristics of the Study Population.

Variables	Level	Overall	Z1	Z2	Z3	Z4	p
N		12783	10760	1300	597	126	
Gender (%)	Female	6265 (49.0)	5403 (50.2)	511 (39.3)	287 (48.1)	64 (50.8)	<0.001
	Male	6518 (51.0)	5357 (49.8)	789 (60.7)	310 (51.9)	62 (49.2)	
Age (mean (SD))		46.68 (16.12)	46.11 (16.08)	48.82 (16.51)	50.39 (15.07)	55.63 (13.22)	<0.001
Race (%)	Mexican American	1952 (15.3)	1762 (16.4)	150 (11.5)	35 (5.9)	5 (4.0)	<0.001
	Non-hispanic black	2833 (22.2)	2342 (21.8)	277 (21.3)	183 (30.7)	31 (24.6)	
	Non-hispanic white	5552 (43.4)	4560 (42.4)	673 (51.8)	247 (41.4)	72 (7.1)	
	Other hispanic	1347 (10.5)	1189 (11.1)	108 (8.3)	43 (7.2)	7 (5.6)	
Other race–- including multi-racial		1099 (8.6)	907 (8.4)	92 (7.1)	89 (14.9)	11 (8.7)	
Education (%)	9–1^1t^h grade	1934 (15.1)	1551 (14.4)	241 (18.5)	109 (18.3)	33 (26.2)	<0.001
	College graduate or above	2976 (23.3)	2618 (24.3)	227 (17.5)	114 (19.1)	17 (13.5)	
	High school graduate	2954 (23.1)	2427 (22.6)	340 (26.2)	148 (24.8)	39 (31.0)	
	Less than ^9t^h grade	1163 (9.1)	965 (9.0)	135 (10.4)	55 (9.2)	8 (6.3)	
	Some college or AA degree	3756 (29.4)	3199 (29.7)	357 (27.5)	171 (28.6)	29 (23.0)	
exposure.BMI (mean (SD))		29.05 (6.80)	29.05 (6.64)	27.36 (5.88)	32.40 (9.18)	30.93 (8.88)	<0.001
VitaminC (mean (SD))		87.27 (101.59)	88.28 (100.95)	83.37 (109.75)	81.10 (96.55)	69.94 (88.74)	0.031
Asthma (%)	No	11043 (86.4)	9496 (88.3)	981 (75.5)	486 (81.4)	80 (63.5)	<0.001
	Yes	1740 (13.6)	1264 (11.7)	319 (24.5)	111 (8.6)	46 (36.5)	
Heart failure (%)	No	12549 (98.2)	10604 (98.6)	1269 (97.6)	563 (94.3)	113 (89.7)	<0.001
	Yes	234 (1.8)	156 (1.4)	31 (2.4)	34 (5.7)	13 (10.3)	
Chronic bronchitis (%)	No	12158 (95.1)	10321 (95.9)	1200 (92.3)	547 (91.6)	90 (71.4)	<0.001
	Yes	625 (4.9)	439 (4.1)	100 (7.7)	50 (8.4)	36 (28.6)	
Dust (%)	No	9943 (77.8)	8451 (78.5)	944 (72.6)	465 (77.9)	83 (65.9)	<0.001
	Yes	2840 (22.2)	2309 (21.5)	356 (27.4)	132 (22.1)	43 (34.1)	
Smoke (%)	No	6935 (54.3)	6177 (57.4)	414 (31.8)	311 (52.1)	33 (26.2)	<0.001
	Yes	5848 (45.7)	4583 (42.6)	886 (68.2)	286 (47.9)	93 (73.8)	
Surgery (%)	No	10041 (78.5)	8519 (79.2)	1017 (78.2)	421 (70.5)	84 (66.7)	<0.001
	Yes	2742 (21.5)	2241 (20.8)	283 (21.8)	176 (29.5)	42 (33.3)	
Tuberculosis (%)	No	12783 (100.0)	10760 (100.0)	1300 (100.0)	597 (100.0)	126 (100.0)	NA

BMI, Body Mass Index. Group Z1 represented the normal lung function group. Group Z2 represents the obstructive ventilatory defect (OVD) group. Group Z3 represented the restrictive ventilatory defect (RVD) group. Group Z4 represents the mixed ventilatory defect (MVD) group.

### 3.2 Association of BMI with lung function

**FIGURE 2:**
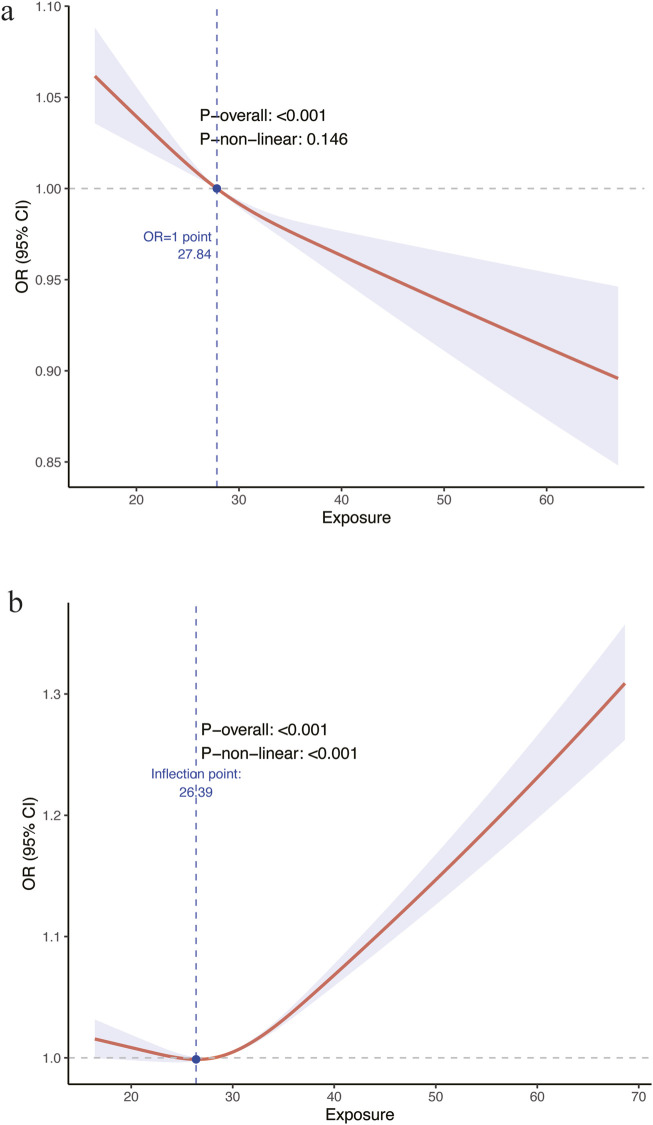
Nonlinear associations between body mass index (BMI) and lung function impairment. **(a)** Associations between BMI and the Z2group. **(b)** Associations between BMI and the Z3 group. OR, odd ratio.

The subjects in the Z2 group were classified into four distinct groups based on BMI values (kg/m^2^): group Q1 (BMI 13.18–24.26), group Q2 (BMI 24.26–27.84), group Q3 (BMI 27.84–32.17), and group Q4 (BMI 32.17–84.87). As shown in Table 2, the relationship between BMI groups and lung function was analyzed using logistic regression models. Model 1 did not adjust for covariates, while Model 2 adjusted for adjusted for age, gender, ethnicity and education. Model 3 further adjusted for vitamin C intake, smoking history, dust inhalation history, asthma, heart failure, and chronic bronchitis. In Model 1, without covariate adjustment, the Q2 group showed a lower risk of lung function impairment compared to the Q1 group, with an odds ratio (OR) of 0.890 (95% CI 0.710–1.174, P = 0.3217). This tendency manifested with greater clarity in the Q3 group (OR = 0.637, 95% CI 0.508–0.800, P = 0.0003) and Q4 group (OR = 0.532, 95% CI 0.418–0.678, P < 0.0001). As shown in Table 2, Similar findings were also observed in model 2 and model 3. Restricted cubic spline analysis did not reveal any significant nonlinear relationships between BMI and lung function (P nonlinear = 0.146, Figure 2a). In instances where BMI exceeds 27.84 kg/m^2^, the odds ratio is less than 1.

**TABLE 2:** Linear regression between BMI and lung function FEV1 Z-scores.

BMI	Model 1		Model 2		Model 3	
	OR (95%CI)	P-value	OR (95%CI)	P-value	OR (95%CI)	Pvalue
Z2 vs. Z1						
Q1	Reference					
Q2	0.890 (0.710, 1.174)	0.3217	0.800 (0.634, 1.011)	0.0698	0.814 (0.655, 1.012)	0.0733
Q3	0.637 (0.508, 0.800)	0.0003	0.543 (0.428, 0.690)	<0.0001	0.554 (0.439, 0.698)	<0.0001
Q4	0.532 (0.418, 0.678)	<0.0001	0.473 (0.371, 0.603)	<0.0001	0.459 (0.365, 0.577)	<0.0001
Z3 vs. Z1						
Q1	Reference					
Q2	0.797 (0.582, 1.093)	0.1656	0.774 (0.555, 1.079)	0.1390	0.774 (0.555, 1.079)	0.1413
Q3	1.114 (0.842, 1.473)	0.4543	1.062 (0.789, 1.430)	0.6926	1.047 (0.777, 1.410)	0.7652
Q4	3.182 (2.346, 4.315)	<0.0001	3.027 (2.219, 4.130)	<0.0001	2.900 (2.708, 4.048)	<0.0001

Model 1: unadjusted; Model 2: adjusted for age, gender, ethnicity and education; Model 3: additional adjustments for vitamin C intake, smoking history, dust inhalation history, asthma, heart failure, and chronic bronchitis.

The subjects in the Z3 group were classified into four distinct groups based on BMI values (kg/m2): Q1 (BMI 14.20–24.47), Q2 (BMI 24.47–28.16), Q3 (BMI 28.16–32.60), and Q4 (BMI 32.60–84.87). In Model 1, without covariate adjustment, the Q2 group showed a lower risk of lung function impairment compared to the Q1 group, with an odds ratio (OR) of 0.797 (95% CI 0.582–1.903, P = 0.1656). Conversely, the Q4 group had a higher risk, with an OR of 3.182 (95% CI 2.346–4.315, P < 0.0001). Similarly, in models 2 and 3, we observed a lower risk of restrictive ventilatory defect in the Q2 group and a higher risk in the Q3 group, although the differences were not statistically significant. In contrast, the risk of restrictive ventilatory defect in the Q4 group remained significantly higher than in Q1, with an OR of 2.900 (95% CI 2.708–4.048, P < 0.0001) in model 3. Restricted cubic spline analysis revealed significant nonlinear relationships between BMI and lung function (P nonlinear <0.001, Figure 2b). The risk of restrictive ventilatory defect decreased with increasing BMI until a tipping point of 26.39 kg/m^2^ was reached. Beyond this threshold, the risk increased significantly with higher BMI.

### 3.3 Subgroup analysis

**FIGURE 3:**
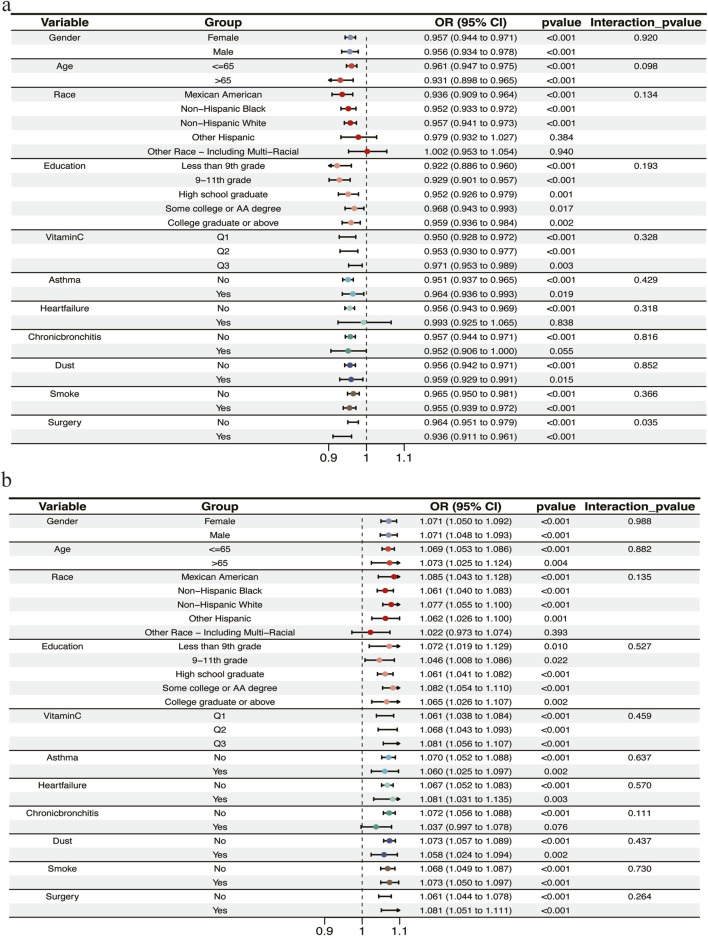
Subgroup analysis of body mass index (BMI) and lung function. **(a)** Subgroup analysis between BMI and the Z2 group. **(b)** Subgroup analysis between BMI and the Z3 group. OR, oddratio.

Subgroup analyses showed consistent negative associations between BMI and obstructive ventilatory defect across every subgroup (Figure 3a). No significant interactions were found for gender (P = 0.920), age (P = 0.098), ethnicity (P = 0.134), education attainment (P = 0.193), vitamin C intake (P = 0.328), asthma (P = 0.429), heart failure (P = 0.318), chronic bronchitis (P = 0.816), dust inhalation history (P = 0.852) or smoking history (P = 0.366). Consequently, these factors did not exert a substantial influence on the relationship between BMI and lung function. While significant interactions were observed for thoracic surgery history (P = 0.035). Compared to the group without a history of thoracic surgery (OR = 0.964, 95% CI 0.951–0.979, P < 0.001), the risk was lower in the group with surgery exposure (OR = 0.936, 95% CI 0.911–0.961, P < 0.001). In contrast, subgroup analyses showed consistent positive associations between BMI and restrictive ventilatory defect across every subgroups (Figure 3b). No significant interactions were found for gender (P = 0.988), age (P = 0.882), ethnicity (P = 0.135), education attainment (P = 0.527), vitamin C intake (P = 0.459), asthma (P = 0.637), heart failure (P = 0.570), chronic bronchitis (P = 0.111), dust inhalation history (P = 0.437), smoking history (P = 0.730) or thoracic surgery history (P = 0.264).

### 3.4 Construction of machine learning-based classification models

As shown in Table 3, the Logistic Regression model achieved an accuracy of 0.4475, a balanced accuracy of 0.4916, a precision of 0.7987, a recall of 0.4475, and an F1-score of 0.5330. The Gradient Boosting model attained an accuracy of 0.8537, a balanced accuracy of 0.3547, a precision of 0.7592, a recall of 0.8357, and an F1-score of 0.7888. The Voting Ensemble model yielded an accuracy of 0.8286, a balanced accuracy of 0.3564, a precision of 0.7582, a recall of 0.8286, and an F1-score of 0.7872.

**TABLE 3:** Comparative analysis of machine learning models.

Model	Accuracy	Balanced accuracy	Precision	Recall	F1 score	Macro AUC
Logistic regression	0.4475	0.4916	0.7987	0.4475	0.5330	0.6637
AdaBoost	0.5099	0.4697	0.7885	0.5099	0.5296	0.6329
SVM	0.6106	0.4012	0.7667	0.6106	0.6708	0.6079
Random forest	0.7974	0.3731	0.7637	0.7974	0.7794	0.6587
Stacking ensemble	0.8108	0.3600	0.7581	0.8108	0.7811	0.6538
Voting ensemble	0.8286	0.3564	0.7582	0.8286	0.7872	0.6645
Gradient boosting	0.8357	0.3547	0.7592	0.8357	0.7888	0.6542

SVM, Support Vector Machine; AUC, Area under the ROC, curve.

The logistic regression model achieved the highest balanced accuracy (49.16%), indicating superior performance in identifying minority classes. In contrast, the gradient boosting model achieved the highest overall accuracy (83.57%) but showed lower balanced accuracy (35.47%), suggesting bias toward the majority class. The voting ensemble demonstrated the best discriminative ability with a macro-average AUC of 66.45%.

**FIGURE 4:**
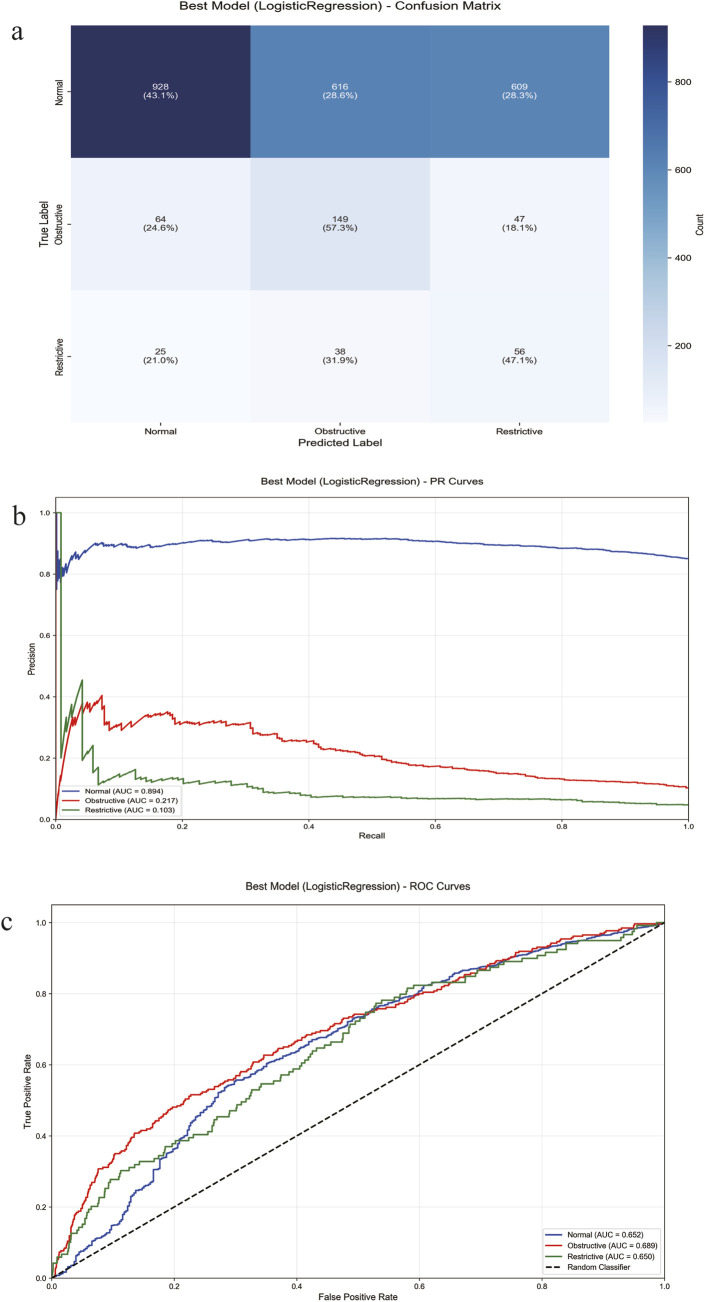
Performance evaluation of the logistic regression model. **(a)** The confusion matrix. **(b)** Precision-recall analysis. **(c)** the Receiver Operating Characteristic (ROC) curve. ROC, Receiver Operating Characteristic Curve. AUC, Area Under the ROC Curve. PR Curve, Precision-Recall Curve. AUPRC, Area Under the Precision-Recall Curve.

The logistic regression model (optimal for balanced accuracy) showed the per-class performance in Table 4. The model successfully identified 57% of obstructive cases and 47% of restrictive cases. However, precision for minority classes remained low. The confusion matrix for the logistic regression model (Figure 4a) revealed specific misclassification patterns. The primary confusion occurred between normal and obstructive classes (41.1% false positives). Precision-recall analysis further highlighted the model’s performance (Figure 4b). The area under the PR curve (AUPRC) was 0.89 (95% CI: 0.87–0.91) for the majority Normal class, indicating strong performance. Conversely, the model achieved a moderate AUPRC of 0.35 (95% CI: 0.31–0.39) for the Obstructive class and a limited AUPRC of 0.18 (95% CI: 0.14–0.22) for the rare Restrictive class, underscoring the challenge of class imbalance for precise discrimination of minority cases. Receiver operating characteristic (ROC) analysis demonstrated moderate discriminative ability across all three classes, with the area under the curve (AUC) being 0.689 (95% CI: 0.652–0.727) for Obstructive, 0.652 (95% CI: 0.630–0.673) for Normal, and 0.650 (95% CI: 0.601–0.699) for Restrictive (Figure 4c).

**TABLE 4:** Per-class performance metrics model.

Class	Precision	Recall	F1-score	Support	AUC
Logistic regression					
Normal	0.91	0.43	0.59	2153	0.6516
OVD	0.19	0.57	0.28	260	0.6893
RVD	0.08	0.47	0.13	119	0.6503
Macro avg	0.39	0.49	0.51	2,532	0.6637

OVD, obstructive ventilatory defect; RVD, restrictive ventilatory defect.

**FIGURE 5:**
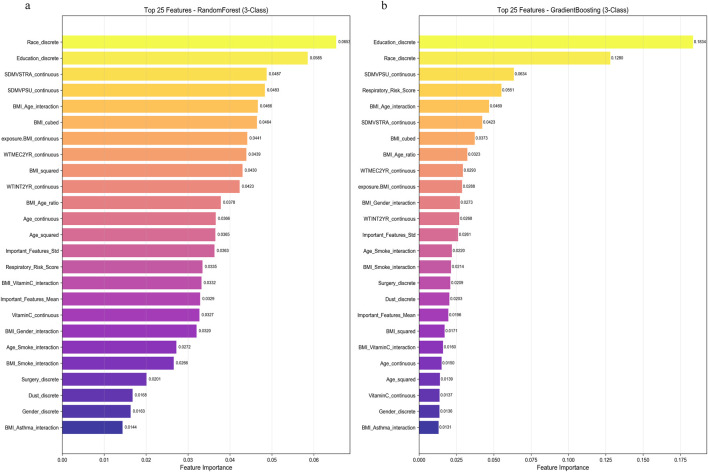
Feature Importance Analysis. **(a)** Feature importance analysis in Random Forest model. **(b)** Feature importance analysis in GradientBoosting model. BMI, body mass index.

Feature importance analysis revealed that BMI-related features collectively accounted for >32% of total importance. Educational attainment and ethnicity ware emerged as important predictor (Figure 5).

## 4 Discussion

This study examined the relationship between BMI and lung function, standardizing lung function measurement through FEV1 Z-scores. A U-shaped relationship between BMI and restrictive ventilatory defect was observed, with both low and high BMI levels serving as risk factors for restrictive ventilatory defect. The lowest risk of lung function impairment was identified at a BMI of 26.39. However, we observed an inverse association between BMI and the risk of an obstructive ventilatory defect, with the risk progressively decreasing as BMI increased. In instances where BMI exceeds 27.84 kg/m^2^, the odds ratio is less than 1. Additionally, male, advanced age, insufficient vitamin C intake, dust exposure, smoking history, asthma, heart failure, chronic bronchitis, and surgery history were found to be significant risk factors for impaired lung function.

BMI serves as a comprehensive health indicator, reflecting body composition aspects such as visceral fat and muscle mass. Numerous studies have explored the complex relationship between BMI and lung function. Some have reported a negative association between abdominal obesity and lung function (Santana et al., 2001; Leone et al., 2009), while others have suggested a positive correlation between BMI and lung function (Çolak et al., 2015). However, these studies were conducted across different countries and regions, focusing mainly on healthy older adult populations. Our study utilized a large sample from the NHANES database (2007–2012), encompassing a broader age range.

The findings reveal a notable non-linear relationship between BMI and restrictive ventilatory defect. The risk of restrictive ventilatory defect decreased significantly as BMI increased, up to a tipping point of approximately 26.39 kg/m^2^. Beyond this threshold, the risk escalated as BMI continued to rise. This non-linear trend was consistently observed across all three models, with the OR change remaining relatively stable. We found moderate overweight (Q2) showed a non-significant trend towards protection, and the association between BMI and lung function was not influenced by potential confounders, such as age. These findings suggest that overweight and mildly obese individuals have a lower risk of impaired lung function. This may be attributed to the secretion of lipocalin from subcutaneous fat, a classical anti-inflammatory agent that reduces airway damage by mitigating inflammation in various cell types through AdipoR1 and R2 signaling mechanisms (Fang and Judd, 2018). While severe obesity (Q4) drastically increased the risk of restrictive impairment. Excessive obesity is recognized as a risk factor for lung function impairment. A cohort study of older Chinese adults found that obesity was linked to reduced FVC (Pan et al., 2017). Similar trends have been observed globally, with a prospective cross-sectional study reporting a negative association between morbid obesity and spirometry variables (Melo et al., 2010). Accumulation of chest wall fat limits lung expansion, while abdominal fat raises the diaphragm, resulting in decreased lung compliance (Brazzale et al., 2015). Enlarged adipocytes and ectopic fat produce and release various metabolic, hormonal, and inflammatory factors that can damage lung parenchyma (Bray et al., 2017). Our findings indicate that the modest protective trend in the Q2 group might be attributed to nutritional benefits, but surpassing this threshold leads to the dominance of traditional harmful mechanisms.

While the risk of obstructive ventilatory defect decreased significantly as BMI increased. In instances where BMI exceeds 27.84 kg/m^2^, the odds ratio is less than 1. This protective effect remained robust after adjusting for a comprehensive set of covariates. This phenomenon aligned with the well-documented “obesity paradox”. The obesity paradox is a seemingly contradictory phenomenon observed in medical research, where overweight or mild obesity (BMI 25–35 kg/m^2^) in certain patient populations with specific diseases is associated with a lower risk of mortality or improved prognosis, while traditionally normal weight (BMI 18.5–25 kg/m^2^) or underweight (BMI <18.5 kg/m^2^) individuals exhibit a higher risk of death (Lennon et al., 2016; Arnold et al., 2016). This paradox is most commonly seen in cardiovascular disease (McAuley et al., 2012; Doehner et al., 2015), chronic obstructive pulmonary disease (COPD) (Gal et al., 2014), and metabolic disorders (Tobias et al., 2014; McAuley and Blair, 2011). Underweight status has been identified as a significant risk factor for lung function impairment. A study in China found that FEV1, FVC, and peak expiratory flow (PEF) were significantly higher in individuals with normal weight compared to underweight individuals (Wang et al., 2017). A cross-sectional study predominantly involving Korean populations also found that being underweight was independently associated with decreased lung function (Do et al., 2019). Several mechanistic hypotheses may explain this finding. First, mechanical factors play a role. In obstructive diseases characterized by airway collapse and loss of lung elastic recoil, the increased body weight and abdominal fat may exert an external pressure on the diaphragm, functionally stenting the peripheral airways and reducing dynamic hyperinflation (Spelta et al., 2017). Additionally, obese individuals may possess greater nutritional and metabolic reserves due to increased fat and/or muscle stores (Poulain et al., 2008), which help counteract inflammatory depletion (Kastorini and Panagiotakos, 2012; Rutten et al., 2013). However, unmeasured factors, such as disease stage and medication use, may have confounded the results.

Subgroup analyses further reinforced the robustness of the primary findings. The negative association between BMI and obstructive defect was remarkably consistent across nearly all subgroups, underscoring the universal nature of this “obesity paradox”. A notable exception was a history of thoracic surgery, where a statistically significant interaction was identified, suggesting a potential modifying role of altered cardiothoracic mechanics on this association. Conversely, the positive association between BMI and restrictive defect was consistently observed without any significant interactions. This indicates that the obesity might be a universal risk factor for restrictive physiology, largely unaffected by other patient characteristics.

Despite we adjusted for a wide array of covariates, the absence of data on disease-specific severity (e.g., GOLD stages for COPD) means we cannot fully rule out this confounding. Therefore, the identified ‘protective’ association between higher BMI and obstructive defect, consistent with the ‘obesity paradox’, may reflect a complex interplay between nutritional status, metabolic reserves, and underlying disease processes, rather than a direct causal, protective effect of excess adiposity itself.

The comparative performance of the three models reveals a critical trade-off between overall accuracy and balanced class identification. The Gradient Boosting and Voting Ensemble models achieved high overall accuracy (>82%) and recall, indicating their proficiency in correctly classifying the majority of instances, predominantly the “Normal” class. However, their substantially lower balanced accuracy (35%) unequivocally signals a pronounced bias toward the majority class, rendering them suboptimal for a clinical context where the identification of pathological cases (obstructive and restrictive defects) is paramount. In contrast, the Logistic Regression model, while yielding a lower overall accuracy, achieved the highest balanced accuracy (49.16%). The detailed analysis of classification performance underscores particular challenges in distinguishing ventilatory defect subtypes. The substantial misclassification between normal and obstructive cases, coupled with the markedly low AUPRC for restrictive defects, highlights the inherent difficulty in achieving precise discrimination of minority classes—a limitation in imbalanced medical datasets. It is noteworthy that feature importance analysis revealed that BMI-related features collectively accounted for over 32% of total importance.

## 5 Strengths and limitations

This study possesses several notable strengths. First, the use of a large, nationally representative sample from the NHANES database substantially enhances the statistical power and generalizability of our findings. Second, we conducted a comprehensive analysis that differentiated between obstructive and restrictive ventilatory defects, revealing distinct and opposing associations with BMI. Third, the robustness of the primary findings was confirmed through extensive multivariable adjustment, rigorous subgroup analyses, and multiple machine learning algorithms, with the logistic regression model demonstrating the most balanced performance for identifying minority classes. Finally, feature importance analysis provided objective, data-driven validation that underscored the paramount role of BMI-related features in predicting lung function status.

Notwithstanding these strengths, several limitations warrant consideration. First, the cross-sectional design precludes the establishment of causal relationships. The persistent concern of reverse causation cannot be fully dismissed; for instance, low BMI may be a consequence rather than a cause of advanced lung disease (e.g., cachexia), while the observed “protective” association in obstructive defects may reflect the complex interplay between underlying disease processes and nutritional status. Second, despite adjusting for a wide array of covariates, residual confounding may persist due to unmeasured factors such as disease-specific severity metrics (e.g., GOLD staging for COPD), physical activity levels, or detailed medication histories. Third, the definition of restrictive ventilatory defect was based solely on FVC without confirmation by total lung capacity (TLC) or diffusion capacity (DLCO) measurements, which may lead to a part of misclassification of ventilatory defects (Ruppel, 2012). Finally, the class imbalance in our dataset posed a significant challenge for the precise classification of minority phenotypes (obstructive and restrictive defects), as evidenced by their lower AUPRC values. This suggests that our models are better suited for screening purposes rather than definitive diagnosis.

## 6 Conclusion

This large-scale, nationally representative study demonstrates a complex and differential relationship between BMI and ventilatory function impairment. Our findings reveal opposing associations: higher BMI confers a protective effect against obstructive ventilatory defects while simultaneously increasing the risk of restrictive ventilatory defects. Notably, the relationship between BMI and restrictive defects follows a U-shaped pattern, with an inflection point at 26.39 kg/m^2^, beyond which risk escalates substantially. These associations remained robust across extensive multivariable adjustments, subgroup analyses, and machine learning validation, with BMI-related features accounting for over 32% of total predictive importance. Future longitudinal studies with detailed pulmonary function measures, imaging data, and mechanistic investigations are warranted to elucidate the underlying pathways and establish temporality.

## Data Availability

All data used in this study were obtained from the publicly available NHANES database (https://www.cdc.gov/nchs/nhanes/index.htm).
